# Association between perinatal factors and future risk for gout—a nested case-control study

**DOI:** 10.1186/s13075-022-02749-2

**Published:** 2022-03-01

**Authors:** Mats Dehlin, Lennart T. H. Jacobsson

**Affiliations:** Department of Rheumatology and Inflammation Research, Institute of Medicine, Sahlgrenska Academy, University of Gothenburg, S-413 45 Göteborg, Sweden

**Keywords:** Gout, Risk factors, Perinatal factors, Birth weight, Epidemiology

## Abstract

**Background:**

Increased level of urate is the strongest risk factor for gout development but since only a minority of hyperuricemics are affected by gout, other pathogenic factors must be considered. Low birth weight is associated with future morbidities causing hyperuricemia, such as diabetes and renal disease. The purpose of this study was to investigate if, and to what extent, maternal and perinatal factors, including birth weight, are associated with future risk of being diagnosed with gout.

**Methods:**

A population-based retrospective nested case-control registry study based on regional and national health care registers in Sweden. All incident cases of gout born in 1973 and onward who had received ≥1 diagnosis of gout from 2000 through 2019 in the region of western Sweden were included. Up to 5 non-gout controls were matched to each case by age, sex, and county at the year of first gout diagnosis. A range of maternal, gestational, and perinatal factors were analyzed for their potential association to future gout development. This included the health of the mother, gestational length, birth weight, number of siblings, and congenital malformations.

**Results:**

Maternal diabetes, any congenital malformation, and being small for gestational age were factors that significantly increased the risk for future gout development, odds ratio (95% CI) 3.1 (1.3 to 7.4) (*p*=0.01), 1.33 (1.04 to 1.7) (*p*=0.02), and 1.75 (1.3 to 2.3) (*p*<.0001), respectively.

**Conclusions:**

In this study, maternal diabetes and being small for gestational age increased the risk for future gout development in young adults. As of today, these conditions are becoming more prevalent and may contribute to the ongoing gout epidemic. These results require both confirmation and further delineation of underlying mechanisms.

## Introduction

Gout is the most common inflammatory joint disease in the world [1] as well as in Sweden [2, 3]. Increased urate level is the strongest risk factor for gout development but since only 15–20% of persons with hyperuricemia (plasma urate >405 μmol/L) develop gout [4] other pathogenic factors must be considered. Gout is an autoinflammatory disease and properties of the innate immune system have been shown to be crucial for the immunological response to urate crystals [4]. The immune system is formed early in life and modulation of it during the perinatal/adolescent period has been suggested as risk factors for other inflammatory joint diseases, such as ankylosing spondylitis [5]. On the other hand, perinatal factors could also be associated with diseases linked with increased levels of urate, such as components of metabolic syndrome, hypertension, hyperlipidemia, and type 2 diabetes [6, 7]. In fact, a recent meta-analysis clearly demonstrated a J-shaped association between birth weight and type 2 diabetes and cardiovascular disease and an inverse relationship with hypertension [7]. Furthermore, low birth weight is associated with end-stage renal disease (ESRD) [8] which results in hyperuricemia and an increased risk of gout. Different malformations may also be associated with hyperuricemia with Lesch-Nyhan probably being the most well-known [9, 10]. Due to these associations, one could expect a J-shaped or inverse association also between birth weight and gout, which has not been previously studied. With this background, we performed an exploratory analysis in the present study to investigate if, and to what extent, a broad range of maternal and perinatal factors, including birth weight, are associated with future risk of being diagnosed with gout.

## Methods

### Study population

All individuals born from 1973 and onward who had received ≥1 main or auxiliary diagnosis of gout at a visit to a physician or a hospitalization from 2000 and through 2019 in western Sweden were identified in the healthcare consumption database VEGA. Up to 5 controls, without a gout diagnosis or a prescription of urate-lowering medication (ATC-code M04) during the same time period, were matched to each case by age, sex, and county at the year of first gout diagnosis. Controls were selected from the Swedish national census register.

### Data sources

#### VEGA

The Western Swedish Health Care Region register (VEGA) was used for the identification of the gout cases. This register contains information about all health care contacts, at both primary and specialized health care units in western Sweden from 2000 onwards. All diagnoses given by physicians are registered according to the Swedish version of the International Classification of Disease (ICD) codes. Since 1997, the 10th version of ICD codes (ICD-10) is used in Sweden.

The comparators were identified from the census register.

#### Medical Birth Register

The Swedish Medical Birth Register (MBR) started in 1973. It includes administrative and medical data related to pregnancy, partum, and postpartum neonatal care [11]. It is compulsory for all health care providers involved in prenatal maternity care, delivery, or neonatal care to report to the register. Data on maternal smoking is only available from 1982 when recording of maternal smoking was initiated.

### Exposure variables

All exposure variables regarding perinatal characteristics were retrieved from the MBR for both cases and controls. The variables assessed were age and marital status of the mother, maternal smoking and body mass index (BMI) at start of pregnancy, maternal stature and parity, mode of delivery (vaginal delivery or caesarian), season of birth, birth weight and gestational age, congenital malformations, number of older siblings, maternal diagnosis of recurrent urinary tract infection, chronic kidney disease, diabetes, epilepsy, asthma/lung disease, inflammatory bowel disease, systemic lupus erythematosus (SLE), and hypertension. The following categorizations were used: age of mother at birth <20 (ref), 20 to 35, and >35 years; maternal stature ≤154 (ref), 155 to 164, 165 to 174, and ≥175 cm; maternal BMI <20 (ref), 20 to 25, 26 to 30, and >30 kg/m^2^; maternal parity 0 (ref) or ≥1 previous births; season of delivery month January to March (ref), April through June, July through August, and October through December; birth weight <2500 (ref), 2500 to 2999, 3000 to 3499, 3500 to 3999, 4000 to 4499, and ≥4500 g; gestational age ≤258 (ref), 259 to 293, and ≥294 days; birth weight for gestational age small (> 2SD below normal weight for gestational age), normal (ref), and large (> 2SD over the normal weight for gestational age) [12]; and the number of older siblings 0 (ref), 1, 2, and ≥3.

### Statistical analysis

Continuous variables are presented as mean ± SD or median (25 and 75 percentile). Categorical variables are presented as numbers and percentages. All variables that showed significant association with gout development univariately were included in a multivariate logistic regression except for maternal smoking and BMI at the start of pregnancy due to large proportions of missing data. Furthermore, the two variables birth weight and birth weight for gestational age both showed a significant association to gout but due to great collinearity between them, we chose to only include the latter in the multivariate analysis. Odds ratios (OR) for developing gout were calculated using conditional logistic regression models. Nominal *p* values < 0.05 were considered statistically significant. Statistical analyses were performed by using SAS version 9.3 (SAS Institute Inc., Cary, NC, USA).

## Results

In total, 1399 cases of gout and 5515 non-gout controls were identified in MBR, 79% with male sex and a median age at first identified gout diagnosis from year 2000 of 34 years (25 percentile 29, 75 percentile 38) (Table 1). A total of 88% of the cases had ≥3 matched controls. There were no significant differences regarding maternal age, maternal stature, and marital status between cases and controls (Table 1) while mean (SD) BMI at the start of pregnancy was significantly higher in gout mothers compared to controls, 22.9 (3.5) and 22.4 (3.3) kg/m^2^, respectively (*p*=0.01) (Table 1). Mean (SD) birth weight was significantly lower in gout cases compared to controls, 3472 (592) versus 3538 (565) g, respectively (*p*=0.0001), while no differences were seen regarding gestational age and number of older siblings (Table 1).

**Table 1:** Perinatal factors for gout cases compared to matched population controls. *Q* quartile, *SD* standard deviation, *n.s.* non-significant

Characteristic	Cases, ***n***=1399	Controls, ***n***=5515	***p*** value
Child sex, male, *n* (%)	1104 (79%)	4324 (78%)	--
Age at first identified gout diagnosis, mean (SD)	33.3 (6.3)	--	--
Age at first identified gout diagnosis, median (Q1, Q3), years	34 (29, 38)	--	--
Year of birth, median (Q1, Q3)	1979 (1975, 1984)	1978 (1975, 1985)	--
Maternal age, years, mean (SD)	26.6 (4.9)	26.8 (4.9)	n.s.
Maternal stature cm, mean (SD)	167 (6.3)	166 (6.2)	n.s.
Maternal BMI kg/m^2^, at start of pregnancy, mean (SD)	22.9 (3.5)	22.4 (3.3)	0.01
Birth weight, grams, mean (SD)	3472 (592)	3538 (565)	0.0001
Gestational age, days, mean (SD)	279 (13.6)	280 (13.1)	n.s.
Number of older siblings, mean (SD)	0.9 (1.0)	0.88 (0.99)	n.s.
Number of older siblings, median (Q1, Q3)	1 (0, 1)	1 (0, 1)	--

### Univariate analysis

In the univariate analysis a significant association with gout was observed for both maternal and off-spring factors. BMI >30 at start of pregnancy and maternal smoking was associated with gout, OR (95% CI) 1.98 (1.09–3.8) (*p*=0.04) and 1.57 (1.25 to 1.96) (*P* <0.0001), respectively, but these results are uncertain due to 70 to 80% missing values in both cases and controls (Table 2).

**Table 2:** Perinatal factors for gout cases compared to matched population controls, with univariate odds ratios, ^a^ Only available from 1982, when recording of maternal smoking was initiated. *OR* odds ratio, *n.s.* non-significant, *CI* confidence interval

Characteristic	Subcategory	Cases, ***n***=1399	Controls, ***n***=5515	Univariate OR (95% CI)	***p*** value
Maternal age groups	<20	75 (5%)	305 (6%)	Ref	
	20–35	1234 (88%)	4800 (87%)	1.05 (0.81–1.4)	0.7
	36–	90 (6%)	410 (7%)	0.9 (0.64–1.3)	0.6
Maternal stature	–154	9 (1%)	50 (1%)	Ref	
	155–164	117 (8%)	553 (10%)	1.18 (0.56–2.5)	0.7
	165–174	209 (15%)	773 (14%)	1.5 (0.7–3.1)	0.27
	175–	34 (2%)	129 (2%)	1.46 (0.66–3.3)	0.35
	Missing	1030 (72%)	4027 (71%)	--	--
Marital status	Married	616 (44)	2466 (45)	Ref	
	Single mother	283 (20)	1071 (19)	1.06 (0.9–1.24)	0.5
	Missing	500 (36)	1978 (36)	--	--
BMI classes, at start, *n* (%)	<20	58 (4%)	273 (5%)	Ref	
	20–25	206 (15%)	835 (15%)	1.16 (0.84–1.6)	0.36
	26–30	38 (3%)	120 (2%)	1.49 (0.9–2.4)	0.09
	>30	16 (1%)	38 (1%)	1.98 (1.04–3.8)	0.04
	Missing	1081 (77%)	4249 (77%)	--	--
Parity	0	584 (42%)	2345 (43%)	Ref	
	≥1	815 (58%)	3170 (57%)	1.03 (0.92–1.16)	0.6
Disease of the mother, *n* (%)	Recurrent urinary tract infection	18 (1.3)	82 (1.5)	0.86 (0.52–1.4)	0.6
	Chronic kidney disease	1 (0.07)	6 (0.11)	--	
	Diabetes	9 (0.64)	12 (0.22)	2.98 (1.25–7.1)	0.01
	Epilepsy	2 (0.1)	4 (0.07)	--	
	Asthma/lung disease	6 (0.4)	12 (0.22)	1.98 (0.7–5.3)	0.2
	Inflammatory bowel disease	2 (0.14)	2 (0.04)	--	
	SLE	0	0	--	
	Hypertension	1 (0.07)	5 (0.09)	--	
Maternal smoking, at start of pregnancy^a^	Yes	159 (11)	480 (9)	1.57 (1.25–1.96)	<.0001
	No	264 (19)	1248 (23)	Ref	
	Missing	976 (70)	3787 (69)		
Season of birth	Month 1–3	358 (26%)	1454 (26%)	Ref	
	Month 4–6	389 (28%)	1503 (27%)	1.05 (0.9–1.2)	n.s.
	Month 7–9	344 (25%)	1351 (25%)	1.03 (0.88–1.2)	n.s.
	Month 10–12	299 (21%)	1183 (21%)	1.03 (0.87–1.2)	n.s.
	Missing	24 (0.4)	9 (0.6)	--	
Mode of delivery, *n* (%)	Vaginal	1353 (97)	5370 (97)	Ref	
	Caesarean (acute and elective)	46 (3)	145 (3)	1.26 (0.9–1.76)	0.17
Birth weight grams, *n* (%)	<2500	73 (5)	200 (4)	Ref	
	2500–2999	185 (13)	595 (11)	0.85 (0.62–1.17)	0.32
	3000–3499	443 (32)	1693 (31)	0.72 (0.54–0.96)	0.02
	3500–3999	456 (33)	1950 (35)	0.64 (0.48–0.85)	0.002
	4000–4499	197 (14)	874 (16)	0.62 (0.45–0.84)	0.002
	≥4500	45 (3)	203 (4)	0.61 (0.4–0.92)	0.02
Birth weight for gestational age, *n* (%)	Small for gestational age	82 (6)	187 (3)	1.77 (1.36–2.3)	<.0001
	Normal for gestational age	1275 (91)	5153 (93)	Ref	
	Large for gestational age	42 (3)	175 (3)	0.97 (0.69–1.37)	0.86
Gestational age, days, *n* (%)	≤258	90 (6)	316 (6)	Ref	
	259–293	1168 (83)	4600 (83)	0.89 (0.7–1.1)	0.3
	≥294	141 (10)	599 (11)	0.83 (0.6–1.17)	0.2
Number of older siblings, *n* (%)	0	582 (42)	2323 (42)	Ref	
	1	512 (37)	2038 (37)	1.003 (0.88–1.15)	n.s.
	2	219 (16)	813 (15)	1.08 (0.9–1.28)	n.s.
	≥3	86 (6)	341 (6)	1.007 (0.78–1.3)	n.s.
Congenital malformation, *n* (%)	Yes	92 (7)	275 (5)	1.34 (1.05–1.7)	0.018
	No	1307 (93)	5240 (95)	Ref	
Five most common malformations, *n* (%)	Urogenital malformation	27 (2)	11 (2)	0.97 (0.63–1.48)	0.88
	Musculoskeletal malformation	12 (1)	40 (1)	1.18 (0.62–2.26)	0.61
	Down’s syndrome	13 (1)	4 (0.1)	12.9 (4.2–39.7)	<.0001
	Cardiovascular malformations	12 (0.8)	27 (0.5)	1.61 (0.8–3.3)	0.2
	Face and throat malformations	6 (0.4)	17 (0.3)	1.39 (0.55–3.54)	0.5

Furthermore, maternal diabetes was significantly related to gout development, OR (95% CI) 2.98 (1.25 to 7.1) (*p*=0.01) (Table 2). Maternal age, stature, marital status, and parity showed no associations to gout. Regarding factors in the off-spring, risk for gout decreased with increasing birth weight with the very lowest risk at ≥4500 g, OR (95% CI) 0.61 (0.4 to 0.92) (*p*=0.02), while being small for gestational age significantly increased risk for gout, OR (95% CI) 1.77 (1.36 to 2.3) (*p*<.0001) (Table 2). Furthermore, any congenital malformation increased the risk of gout, OR (95% CI) 1.34 (1.05 to 1.7) (*p*=0.018), and specifically, Down’s syndrome showed a great risk increment for gout development, OR (95% CI) 12.9 (4.2 to 39.7) (*p*<.0001) (Table 2). Season of birth, mode of delivery, gestational age, and number of older siblings showed no association with being diagnosed with gout in the future.

### Multivariate analysis

In the multivariate analysis, the findings from the univariate analysis were confirmed (Table 3).

**Table 3:** Univariate and multivariate logistic regression with stepwise addition for probability to develop future gout. *OR* odds ratio, *CI* confidence interval

	Univariate, odds ratio, 95% CI	*p* value	Multivariate odds ratio, 95% CI model 1	*p* value	Multivariate odds ratio, 95% CI model 2	*p* value
**Maternal diabetes**						
No	Ref		Ref		Ref	
Yes	2.98 (1.25–7.1)	0.01	3.05 (1.27–7.3)	0.01	3.1 (1.3–7.4)	0.01
**Birth weight for gestational age**						
Small for gestational age	1.77 (1.36–2.3)	<.0001	1.77 (1.4–2.3)	<.0001	1.75 (1.3–2.3)	<.0001
Normal for gestational age	Ref		Ref		Ref	
Large for gestational age	0.97 (0.69–1.37)	0.86	0.92 (0.7–1.3)	0.6	0.92 (0.6–1.3)	0.6
**Congenital malformation**						
No	Ref				Ref	
Yes	1.34 (1.05–1.7)	0.018			1.33 (1.04–1.7)	0.02

Model 1: maternal diabetes and birth weight for gestational age

Model 2: maternal diabetes, birth weight for gestational age, and congenital malformation

Maternal diabetes, congenital malformation, and small for gestational age all significantly and independently increased the risk for future gout development, OR (95% CI) 3.1 (1.3 to 7.4) (*p*=0.01); 1.33 (1.04 to 1.7) (*p*=0.02); and 1.75 (1.3 to 2.3) (*p*<.0001), respectively (Table 3).

## Discussion

In this retrospective nested case-control study, we examined perinatal factors and their association to being diagnosed with gout in the future. Maternal diabetes, being born with any congenital malformation, and being small for gestational age all increased the risk for future gout.

### Maternal diabetes

In the current study, maternal diabetes was strongly associated with future gout. There are numerous reports on exposure in utero to maternal diabetes influencing long-term metabolic outcomes in the offspring, with a higher risk of obesity and type 2 diabetes [13]. In spite of this, the possible mechanisms behind the exposure to intrauterine hyperglycemia have not been understood. Maternal pre-pregnancy BMI has been proposed as an alternative mechanism [14, 15], through the inheritance of maternal (parental) obesity. Unfortunately, in our study, data on maternal weight is to a large extent missing. The prevalence of diabetes in mothers with gout-offspring, based on a low number of diabetic mothers, was in the current study relatively high, close to 0.7%. However, over the last decades all types of diabetes in pregnancy have increased in both Sweden and worldwide [16, 17], especially type 2 diabetes, thus possibly increasing the importance of maternal diabetes in more recent birth cohorts and possibly further adding to the ongoing gout epidemic.

### Small for gestational age/birth weight—Barker hypothesis

Birth weight was significantly negatively associated with future gout in our study, with the highest risk seen in weights below 2500 g, which is the WHO definition for low birth weight (LBW) [18]. Today, LBW is in the majority of cases related to premature birth [19], <37 weeks of gestation, but in the current study, there was no difference in gestational length between cases and controls. Since the incidence of preterm birth has increased over recent decades, partly due to advances in prenatal care, increased number of multiple births associated with the use of in vitro fertilization, and the increasing age of women giving birth [20] this may be an association of increasing importance.

The Barker hypothesis, proposed by Barker and colleagues more than three decades ago, states that intrauterine starvation leads to changed fetal developmental programming, abnormal organogenesis, LBW, and through this an increased risk for cardiovascular disease, diabetes, obesity [21], and end-stage renal disease [9] later in life. Whether the association with gout that we describe is mediated through these comorbidities or if birth weight may have a specific effect on urate levels or development of clinical gout itself can not be determined from our results. Nevertheless, it may be hypothesized that actions aimed to reduce the number of off-spring born underweight may, in addition to reducing the risk for the morbidities mentioned above, also reduce the risk for gout [7].

### Congenital malformation

There are no previous reports on any associations between congenital malformations and future gout. However, malformation is a wide term and spans from missing a toe to advanced genetic disturbances such as Down syndrome. The association for future gout to Down syndrome in this study (based on few exposed subjects) has not been described earlier except for some case reports. A possible underlying mechanism could be the increased frequency of the metabolic syndrome in individuals with trisomy 21, such as dyslipidemia, diabetes, and obesity [22]. Down syndrome occurs in 12–14 per 10,000 live births in Sweden [23] and is accordingly strongly overrepresented in the current gout study cohort with 13 cases in 1500 individuals.

### Siblings

We found no association between number of older siblings and risk for gout and neither did Wallace et al in their study from 1967 [24]. Having older siblings has been used as a proxy for infections during childhood and adolescence with possible effects on the immune system, the higher number of older siblings the higher exposure to infections. One explanation could be that the innate immune system, involved in the pathological inflammatory processes in gout, is inherently more stable compared to the adaptive immune system. However, the effect of adolescent infections on risk of other, more autoimmune, arthritic diseases has yielded conflicting results. A previous Swedish study on rheumatoid arthritis has not shown any association to the number of siblings [25] while a statistically significant increased risk for developing ankylosing spondylitis was observed in those having older siblings [26].

### “Young gout”

What is a typical gout patient? It may differ around the world but in western Sweden, it is a male aged 65 or above with multiple comorbidities. In the current study, data on perinatal factors was retrieved from the MBR which started in 1973 which rendered our oldest cases of gout 46 years of age. Thus, it is not possible to generalize these findings to the whole gout population, and studies are needed both to confirm our results in subjects with onset of gout at a younger age and with extended analyses in higher age groups.

### Strengths and limitations

Some possible limitations in our study should be acknowledged. First, the identification of the patients with gout was based on ICD codes, which may have led to some misclassification bias. However, in previous validation studies, the ICD-coded definition of gout was found to have a high positive predictive value for fulfilling the various classification criteria for gout [27, 28]. Second, the lack of data for maternal BMI, smoking, and socioeconomic status are three very important factors for offspring outcome. Third, the low age of the gout cohort decreases generalizability, in particular to gout in more common ages of disease onset.

There are some strengths to our study. First, it is population based including all gout cases in western Sweden born in 1973 and later which reduces the risk of selection bias. Second, patients were identified from both primary and specialized health care, which covers all the different phenotypes of gout, from mild to severe disease. Third, our data was retrieved from independent national compulsory registers with prospectively collected data.

## Conclusion

We suggest that maternal diabetes and small for gestational age are perinatal factors that increase the risk for future gout development in young adults. Since these factors are increasing in occurrence, they may contribute to the ongoing gout epidemic and thus require both confirmation and further delineation of underlying mechanisms.

## Data Availability

The datasets analyzed during the current study are available from the corresponding author on reasonable request.

## Abbreviations

ESRD: End-stage renal disease
VEGA: The Western Swedish Health Care Region register
ICD: The International Classification of Disease
MBR: The Swedish Medical Birth Register
BMI: Body mass index
SLE: Systemic lupus erythematosus
LBW: Low birth weight
